# Assessment of different thresholds of birthweight discordance for early neonatal outcomes: retrospective analysis of 2348 twin pregnancies

**DOI:** 10.1186/s12884-022-04417-4

**Published:** 2022-02-01

**Authors:** Shaoxin Ye, Dazhi Fan, Pengsheng Li, Gengdong Chen, Jiaming Rao, Huishan Zhang, Zixing Zhou, Jinping Feng, Caihong Luo, Xiaoling Guo, Zhengping Liu, Dongxin Lin

**Affiliations:** 1 Foshan Institute of Fetal Medicine, Affiliated Foshan Maternity & Child Healthcare Hospital, Southern Medical University, 11 Renminxi Road, Foshan, 528000 Guangdong China; 2 Department of Obstetrics, Affiliated Foshan Maternity & Child Healthcare Hospital, Southern Medical University, Foshan, 528000 Guangdong China

**Keywords:** Birthweight discordance, Twin pregnancies, Neonatal outcome, Dose-response relationship, Predictive accuracy

## Abstract

**Background:**

The optimal threshold of birthweight discordance (BWD) remains controversial. This study aimed to evaluate the associations between BWD at different thresholds and early neonatal outcomes and to assess their predictive accuracy.

**Methods:**

This was a retrospective cohort study using a birthweight data with the chorionicity information of 2348 liveborn twin pairs at a gestational age of ≥26 weeks, from 2012 to 2018. The percentage of BWD was calculated by dividing the actual birthweight difference by the weight of the larger twin and multiplying by 100. Outcomes of interest included neonatal intensive care unit (NICU) admission, neonatal respiratory distress syndrome (NRDS), ventilator support and a composite outcome combining major morbidities and neonatal death. Logistic regression models were performed to estimate the association between neonatal outcomes and BWD with different thresholds (≥15.0%, ≥20.0%, ≥25% and ≥ 30%). Generalized estimated equation (GEE) models were used to address intertwin correlation. Restrictive cubic spline (RCS) models were established to draw the dose-response relationship between BWD and the odds ratios of outcomes. Clustered receiver operating characteristic (ROC) curve analyses were performed to assess the predictive accuracy.

**Results:**

Of 2348 twin pairs, including 1946 dichorionic twin pairs and 402 monochorionic twin pairs, BWD was significantly associated with NICU admission, regardless of the thresholds used. The incidence of NRDS, ventilator support and the composite outcome were significantly higher when a threshold of ≥20% or greater was chosen. The dose-response relationship showed nonlinear growth in the risk of adverse neonatal outcomes with increasing BWD. ROC analyses showed a low significant AUROC of 0.569 (95% CI: 0.526–0.612) for predicting NICU admission but no significant AUROCs for predicting other outcomes. A BWD of ≥30% provided a moderate increase in the likelihood of NICU admission [positive likelihood ratio (LR^+^) = 5.77].

**Conclusion:**

Although BWD is independently associated with adverse neonatal outcomes, it is not a single predictor for neonatal outcomes given the weak discriminative ability to predict neonatal outcomes. A cutoff of 30% is more practical for risk stratification among twin gestations.

**Supplementary Information:**

The online version contains supplementary material available at 10.1186/s12884-022-04417-4.

## Background

Twin pregnancies are well-known to be at increased risks of perinatal mortality and morbidity compared to singletons [1, 2]. Growth discordance is a unique term used among pregnancies with twins or higher orders, and is usually monitored using estimated fetal weight (EFW) based on ultrasound examination in clinical practice [3]. Discordant growth to a certain degree may be inevitable, given that mild discordance may represent a normal variation between twin fetuses or an adaptation to limited intrauterine space [4]. However, severe growth discordance to a significant degree represents abnormal fetal growth and is associated with poor perinatal outcomes [5–7].

The optimal cutoff of significant discordant growth, beyond which a twin pregnancy may experience increased risks of adverse outcomes, remains controversial. Several thresholds are adopted in current literature and clinical practice ranging from 15 to 30%. Among these threshold values, 20 and 25% are more commonly used [5, 6, 8–11]. A cutoff of 20% to define significant growth discordance is suggested by the American College of Obstetricians and Gynecologists [12] whereas a cutoff of 25% is suggested by the National Institute for Health and Care Excellence (NICE) guidance [13]. Previous studies used different cutoffs to evaluate the association between growth discordance and perinatal outcomes. However, the robustness of these results could be impeded by several limitations, including small sample size; lack of chorionicity information; use of EFW via ultrasound screening, which is less accurate; and ignorance of intertwin correlation in analyses [14–18]. Another question is whether discordant growth is clinically useful as a predictor of adverse outcomes. To the best of our knowledge, only a small number of studies assessed the predictive accuracy of growth discordance for predicting adverse outcomes and explored the optimum cutoff based on receiver operating characteristic (ROC) analyses [5, 9, 19, 20].

In the current study, we aimed to evaluate the associations between neonatal outcomes and BWD using different thresholds, based on birthweight data with chorionicity information. Additionally, we aimed to assess the ability of BWD to predict these outcomes.

## Methods

### Study design

This retrospective cohort study was performed at a tertiary hospital in Foshan, China, during the period from January 2012 to December 2018. All electric medical records of eligible subjects were systematically reviewed. This study was approved by the ethics committee of Southern Medical University Affiliated Maternal & Child Health Hospital of Foshan, and informed consent from the patients was waived due to the retrospective design (ethics approval number: FSFY-20180903). All methods were performed in accordance with relevant guidelines and regulations.

### Inclusion and exclusion criteria

**Fig. 1:**
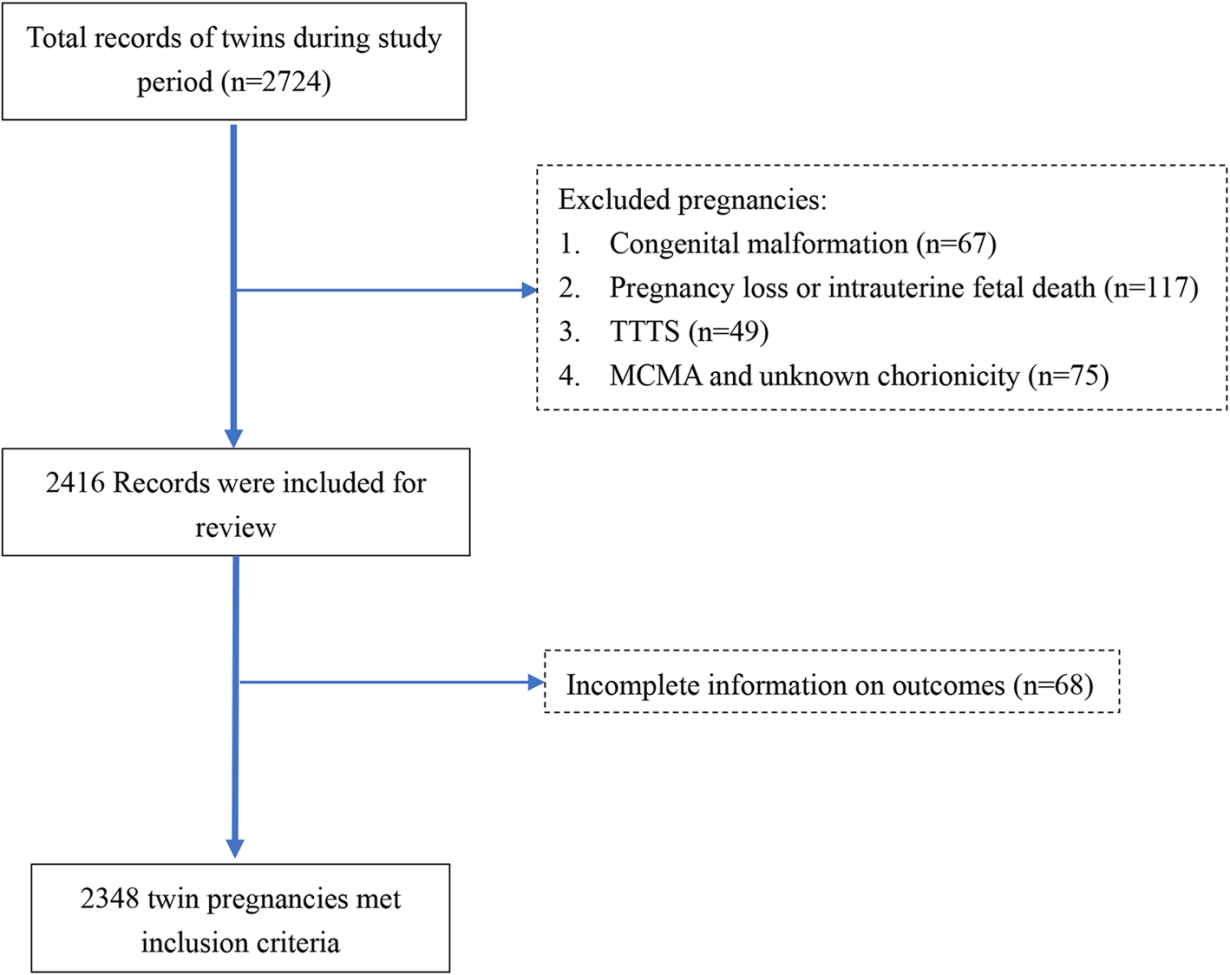
Flow diagram of the inclusion of subjects in analysis

Twin pregnancies with both liveborn fetuses at a gestational age of ≥26 weeks were considered for inclusion. The exclusion criteria included congenital anomalies (except for persistent ductus arteriosus in cases of preterm birth), twin-to-twin transfusion (TTTS), fetal loss before 26 weeks and intrauterine death, monoamniotic twins and pregnancies with unknown chorionicity (Fig. 1).

### Data collection

The following information was collected: maternal age, ethnicity, marital status, chorionicity, parity, use of assisted reproductive technology (ART), obstetric complications (e.g., gestational diabetes mellitus and pregnancy-related hypertensive disorder), mode of delivery, gestational age at delivery, neonatal sex and birthweight. Chorionicity was determined at the first sonographic examination and was confirmed by placental pathologic findings after birth, if available. For IVF/ICSI pregnancies the gestational age was calculated from the date of embryo transfer (+ 14 days). For spontaneous pregnancies, the gestational age was calculated based on the last menstrual period and was further confirmed by sonography in the first trimester. The percentage of birthweight discordance (BWD) was calculated by dividing the actual birthweight difference by the weight of the larger twin and multiplying by 100. Small for gestational age (SGA) was defined when the birth weight was below the 10th percentile for gestational age and sex based on twin birth weight curves in Chinese twins [21, 22].

### Outcomes of interest

The primary outcome of interest was neonatal intensive care unit (NICU) admission, whereas secondary outcomes included neonatal respiratory distress syndrome (NRDS) and ventilator support. We also defined a composite outcome as any occurrence of the following major morbidities and mortality: hypoxic-ischemic encephalopathy (HIE), bronchopulmonary dysplasia (BPD), necrotizing enterocolitis (NEC), intracranial hemorrhage (ICH), culture-proven sepsis and neonatal death within 28 days after birth.

### Statistical analysis

All statistical analyses were performed using Stata, version 15.1 (Stata Corp, College Station, TX). Baseline characteristics were compared between five BWD groups determined based on the selected thresholds (< 15.0, 15.0–19.9%, 20.0–24.9%, 25.0–29.9% and ≥ 30.0%). Continuous variables are presented as the mean ± standard deviation (SD), whereas categorical variables are presented as frequencies with their accompanying percentages. Differences were examined by using analysis of variance (ANOVA) for continuous variables and the chi-square test or Fisher’s exact test for categorical variables.

To assess the association between the neonatal outcomes and BWD defined by the selected cutoffs, univariable logistic regression models were first performed to obtain crude odds ratios (ORs) and 95% confidence intervals (CIs). Multivariable logistic regression models were further performed to control for confounders. In this procedure, two multivariable models were performed. Model 1 adjusted for the use of ART, nulliparity, chorionicity and gestational age. Model 2 further adjusted for SGA status. Generalized estimated equation (GEE) analysis was utilized to account for the within-pair effect. Logistic regression models stratified by chorionicity and larger/smaller twins were also performed. Restrictive cubic spline (RCS) models were conducted to establish the dose-response relationship between continuous BWD and the adjusted ORs of neonatal outcomes. Clustered ROC curve analysis, adjusted for confounders, was performed to determine the optimal threshold of BWD and to assess its predictive accuracy. The sensitivity, specificity, positive/negative predictive value (PPV and NPV), and positive/negative likelihood ratio (LR^+^ and LR^−^) of each cutoff were calculated. An LR^+^ of > 10 indicates a significant increase in the likelihood of disease, whereas an LR^+^ ranging from 5 to 10 and below 5 indicates a moderate and minimal increase, respectively [5]. The adjusted area under the ROC curve (AUROC) was also calculated. Confidence interval estimates were obtained from 1000 bootstrap replications. An AUROC curve of 0.5 indicates no discrimination, whereas an AUROC curve of 1.0 indicates perfect discrimination. All *P*-values were two-sided at a significance level of 0.05.

## Results

A total of 2348 pairs of twins, including 1946 dichorionic twin pairs and 402 monochorionic twin pairs, met the inclusion criteria. The numbers of pregnancies with BWDs of < 15.0%, 15.0–19.9, 20.0–24.9%, 25–29.9% and ≥ 30.0% were 1799, 251, 143, 85 and 75, respectively. Characteristics based on BWD groups are shown in Table 1. Significant differences in chorionicity, nulliparity, pregnancy-related hypertensive disorder and SGA were noted between the five BWD groups (all *P* < 0.05). Twins with severe BWD (≥30.0%) were delivered at earlier gestational ages than those with lower-level BWD (*P* = 0.003).

**Table 1:** Comparison of characteristics between twin pregnancies with different degrees of growth discordance

Variables	< 15.0% (*n* = 1799)	15.0–19.9% (*n* = 251)	20.0–24.9% (*n* = 143)	25.0–29.9% (*n* = 85)	≥30.0% (*n* = 70)	*P*-value
Advanced maternal age (≥35 years)	376 (20.9)	58 (23.1)	27 (18.9)	13 (15.3)	17 (24.3)	0.526
Han ethnic	1767 (98.2)	248 (98.8)	140 (97.9)	84 (98.8)	69 (98.6)	0.965*
Not married	38 (2.1)	6 (2.4)	4 (2.8)	4 (4.7)	1 (1.4)	0.499*
Monochorionic pregnancies	305 (17.0)	41 (16.3)	25 (17.5)	10 (11.8)	21 (30.0)	0.039
ART conceived pregnancies	1150 (63.9)	177 (70.5)	101 (70.6)	58 (68.2)	41 (58.6)	0.094
Nulliparity	1213 (67.4)	179 (71.3)	113 (79.0)	70 (82.4)	51 (72.9)	0.002
Cesarean section	1755 (97.6)	249 (99.2)	140 (97.9)	84 (98.8)	67 (95.7)	0.306
Gestational age at delivery (weeks)	35.6 ± 2.1	35.3 ± 2.3	35.6 ± 2.0	35.0 ± 2.5	31.7 ± 6.4	0.003
Gestational diabetes mellitus	341 (19.0)	59 (23.5)	26 (18.2)	13 (15.3)	13 (18.6)	0.405
Pregnancy-related hypertensive disorder	164 (9.1)	34 (13.6)	23 (16.1)	11 (12.9)	20 (28.6)	< 0.001
Same sex	1162 (64.6)	160 (63.8)	79 (55.2)	50 (58.8)	49 (70.0)	0.131
SGA in either twin	129 (7.2)	45 (17.9)	48 (33.6)	51 (60.0)	62 (88.6)	< 0.001

^*^Fisher’s exact test

**Fig. 2:**
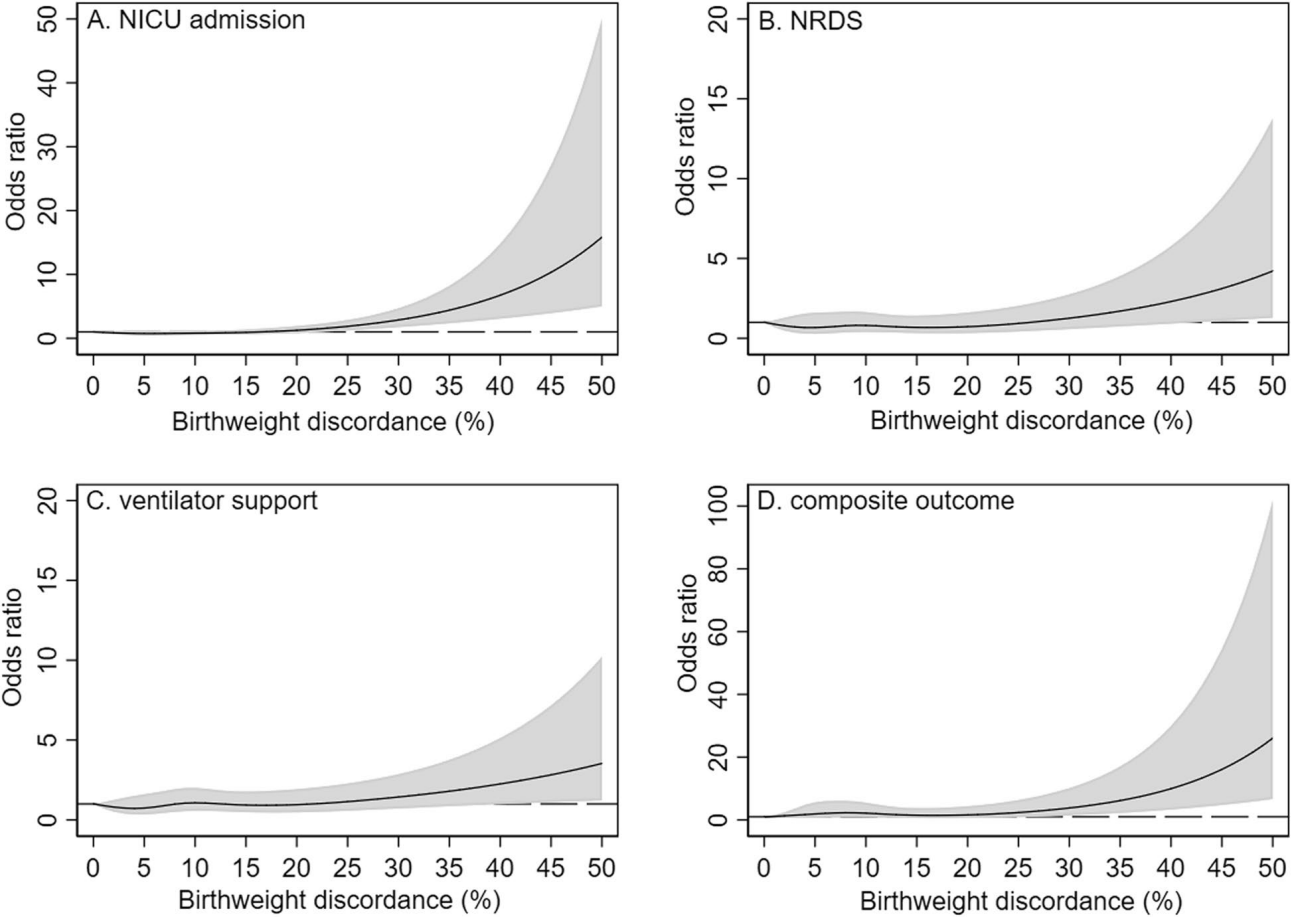
The dose-response relationship between birthweight discordance and neonatal outcomes. Dashed lines represent the reference line (OR = 1.0); gray bands represent 95% confidential intervals

The ORs and 95% CIs for different BWD thresholds obtained from univariable and multivariable logistic regressions are shown in Table 2. In general, we found that twins with BWD were at significantly increased risk of NICU admission irrespective of the thresholds. Overall, there was an increasing trend in the odds ratios of NICU admission with increasing BWD cutoffs. Although the adjusted ORs became lower when the SGA status was further controlled than before (Model 2), the values remained statistically significant. In terms of NRDS, significantly increased adjusted ORs were observed when a cutoff of 20% or greater was used. Similarly, twins with a BWD of ≥20% or greater were more likely to have ventilator support. After further adjusting for SGA status, however, the risk was only found in twins with BWD ≥30% (aOR: 2.39; 95% CI: 1.25–4.57). Twins with BWD of ≥20% were also had an increased possibility experiencing composite outcomes. The results stratified by chorionicity are presented in Table S1. Overall, MCDA (*n* = 804) and DCDA twins (*n* = 3892) with BWD were associated with NICU admission irrespective of the cut-offs used. However, BWD was not associated with other outcomes in MCDA twins, but it was associated with other outcomes in DCDA twins when specific cutoffs were chosen (20, 25 and 30%). The results stratified by the larger and smaller twins are shown in Table S2. Increased adjusted ORs of NICU admission were found in the larger twins when a cutoff of 20% or greater was used, whereas increased adjusted ORs were found in the smaller twin regardless of the cutoffs used. Furthermore, the larger twins in BWD pairs were associated with NRDS when a cutoff of 20% was used, whereas the smaller twins in BWD pairs were not associated with NRDS, regardless of the cutoff was used. The smaller twins in BWD pairs were more likely to have ventilator support when a cutoff of 30% was used and to have composite outcome when a cutoff of 25% or greater was used. In contrast, the larger twins in BWD pairs were not associated with ventilator support or composite outcome regardless of the cutoffs used. The RSC models, in which BWD was regarded as a continuous variable, showed nonlinear relationships between BWD and all the outcomes with adjustment for the use of ART, chorionicity, nulliparity and gestational age at delivery (Fig. 2). The odds ratio of NICU admission began to increase at a BWD of 20% (Fig. 2A). For NRDS and ventilator support, the curves were flatter. These odds ratios became significant at a BWD ≥40% given the overlaps between the band of the confidential interval and the reference line (Fig. 2B and C). Regarding the composite outcome, the risk increased at a BWD of approximately 25% (Fig. 2D). Stratified analyses showed different relationships between the odds ratios of NICU admission and BWD regrading DCDA/MCDA twins and smaller/larger twins (Fig. S1 and S2). The ORs were gradually higher among MCDA twins than DCDA twins, and higher among smaller twin than larger twins.

**Table 2:** Association between neonatal outcomes and birthweight discordance by different cutoffs

Outcomes	Crude OR	*P*-value	Adjusted OR^a^	*P*-value	Adjusted OR^b^	*P*-value
NICU admission						
≥ 15%	1.93 (1.62–2.30)	< 0.001	1.84 (1.47–2.32)	< 0.001	1.54 (1.23–1.93)	< 0.001
≥ 20%	2.55 (2.03–3.21)	< 0.001	2.71 (2.03–3.64)	< 0.001	1.83 (1.37–2.44)	< 0.001
≥ 25%	3.54 (2.56–4.91)	< 0.001	3.58 (2.37–5.41)	< 0.001	2.21 (1.50–3.26)	< 0.001
≥ 30%	6.05 (3.49–10.51)	< 0.001	6.33 (3.2–12.53)	< 0.001	2.44 (1.37–4.36)	0.002
NRDS						
≥ 15%	1.31(0.97–1.77)	0.077	1.10 (0.73–1.65)	0.655	1.06 (0.70–1.60)	0.780
≥ 20%	1.81 (1.28–2.56)	0.001	2.24 (1.41–3.56)	0.001	2.20 (1.38–3.51)	0.001
≥ 25%	2.39 (1.57–3.62)	< 0.001	2.39 (1.38–4.16)	0.002	2.32 (1.32–4.09)	0.004
≥ 30%	2.55 (1.42–4.56)	0.002	2.41 (1.15–5.03)	0.020	2.30 (1.09–4.87)	0.029
Ventilator support						
≥ 15%	1.40 (1.05–1.86)	0.020	1.22 (0.86–1.72)	0.273	1.12 (0.78–1.61)	0.532
≥ 20%	1.59 (1.13–2.24)	0.007	1.67 (1.11–2.51)	0.014	1.51 (0.98–2.33)	0.059
≥ 25%	2.14 (1.42–3.22)	< 0.001	1.87 (1.14–3.07)	0.013	1.66 (0.99–2.80)	0.057
≥ 30%	2.77 (1.61–4.77)	< 0.001	2.66 (1.43–4.96)	0.002	2.39 (1.25–4.57)	0.009
Composite outcome						
≥ 15%	1.44 (0.88–2.33)	0.145	1.37 (0.82–2.27)	0.228	1.28 (0.76–2.14)	0.355
≥ 20%	1.77 (1.01–3.09)	0.045	2.11 (1.18–3.77)	0.012	1.92 (1.06–3.49)	0.032
≥ 25%	2.80 (1.51–5.18)	0.001	2.88 (1.50–5.56)	0.002	2.58 (1.30–5.13)	0.007
≥ 30%	3.90 (1.82–8.33)	< 0.001	5.24 (2.32–11.86)	< 0.001	4.60 (1.95–10.84)	< 0.001

^a^Model 1 adjusted for nulliparity, use of ART, chorionicity and gestational age

^b^Model 2 adjusted for nulliparity, use of ART, chorionicity, gestational age and SGA status

**Fig. 3:**
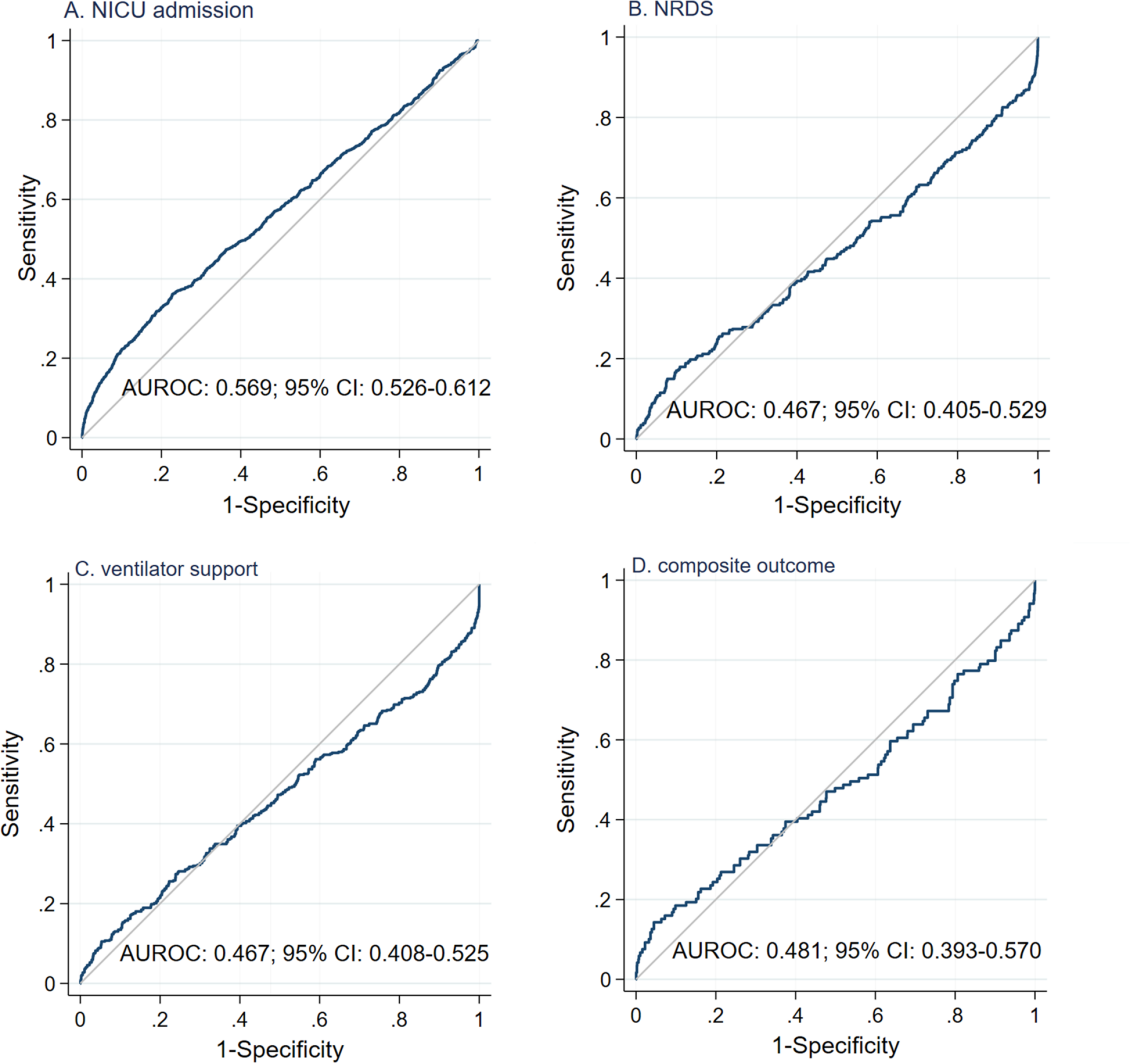
ROC curve analysis of birthweight discordance for the prediction of neonatal outcomes

The clustered ROC curve analyses showed that BWD had a significant AUROC of 0.569 (95% CI: 0.526–0.612) for predicting NICU admission but insignificant AUROCs for predicting NRDS, ventilator support and composite outcome (Fig. 3). In the stratified analysis, BWD had significant AUROCs for predicting NICU admission among DCDA (AUROC: 0.545; 95% CI: 0.510–0.582) and MCDA (AUROC: 0.676; 95% CI: 0.612–0.740) twins (Fig. S3 and S4) but did not have significant AUROCs for predicting other outcomes. BWD had a significant AUROC for predicting NICU admission among the smaller twins (AUROC: 0.615; 95% CI: 0.570–0.659) but not among the larger twins (AUROC: 0.525; 95% CI: 0.403–0.533) (Fig. S5 and S6). We further calculated the diagnostic indices of selected thresholds of BWD for NICU admission, as shown in Table 3. Overall, the selected cutoffs showed a high specificity (ranging from 81.7 to 99.0%) but a low sensitivity (ranging from 5.6 to 30.1%) in predicting NICU admission. A BWD of ≥30% had an LR^+^ of 5.77, indicating a moderate increase in the likelihood of NICU admission. In contrast, other thresholds had a low LR^+^ less than 5.0. In the stratified analyses, a BWD of ≥30% had an LR^+^ greater than 5.0 in the subgroups of MCDA twins and smaller twins but had an LR^+^ less than 5.0 in the subgroups of DCDA twins and larger twins.

**Table 3:** Sensitivity, specificity, positive and negative predictive value (PPV and NPV), and positive and negative likelihood ratio (LR^+^ and LR^−^) of different cutoffs of birthweight discordance for prediction of NICU admission

Cut-offs	Sensitivity	Specificity	PPV	NPV	LR^+^	LR^−^
All twins						
≥ 15%	30.1 (28.1–32.2)	81.7 (80.2–83.2)	55.6 (52.6–58.6)	60.6 (59.0–62.2)	1.65 (1.49–1.83)	0.85 (0.83–0.88)
≥ 20%	18.6 (16.9–20.4)	91.8 (90.7–92.8)	63.3 (59.2–67.1)	59.7 (58.2–61.2)	2.26 (1.94–2.65)	0.89 (0.87–0.91)
≥ 25%	10.9 (9.6–12.4)	96.7 (95.9–97.3)	71.3 (65.9–76.2)	58.8 (57.3–60.3)	3.27 (2.57–4.15)	0.92 (0.91–0.94)
≥ 30%	5.6 (4.7–6.7)	99.0 (98.6–99.3)	81.4 (73.8–87.3)	58.0 (56.5–59.4)	5.77 (3.78–8.80)	0.95 (0.94–0.96)
MCDA twins						
≥ 15%	34.8 (30.3–39.6)	87.5 (83.7–90.6)	75.3 (68.5–81.0)	55.2 (51.2–59.2)	2.79 (2.08–3.75)	0.74 (0.69–0.80)
≥ 20%	22.0 (18.1–26.3)	94.8 (92.0–96.7)	82.1 (73.5–88.5)	52.7 (48.9–56.5)	4.23 (2.66–6.72)	0.82 (0.78–0.87)
≥ 25%	13.4 (10.3–17.1)	98.4 (96.5–99.4)	90.3 (79.5–96.0)	51.1 (47.4–54.7)	8.58 (3.74–19.68)	0.88 (0.85–0.91)
≥ 30%	9.3 (6.8–12.6)	99.2 (97.5–99.8)	92.9 (79.4–98.1)	50.1 (46.5–53.7)	11.95 (3.72–38.34)	0.91 (0.89–0.94)
DCDA twins						
≥ 15%	28.9 (26.7–31.2)	80.8 (79.1–82.4)	51.4 (48.1–54.7)	61.7 (59.9–63.5)	1.50 (1.34–1.68)	0.88 (0.85–0.91)
≥ 20%	17.7 (15.9–19.7)	91.3 (90.0–92.4)	58.9 (54.3–63.3)	61.2 (59.5–62.8)	2.03 (1.72–2.41)	0.90 (0.88–0.92)
≥ 25%	10.3 (8.8–11.9)	96.4 (95.5–97.1)	66.5 (60.2–72.3)	60.4 (58.8–62.0)	2.82 (2.18–3.64)	0.93 (0.92–0.95)
≥ 30%	4.7 (3.7–5.8)	99.0 (98.5–99.3)	76.5 (66.7–84.3)	59.6 (58.0–61.1)	4.63 (2.91–7.35)	0.96 (0.95–0.97)
Larger twins						
≥ 15%	27.2 (24.4–30.1)	79.3 (77.0–81.3)	47.5 (43.3–51.8)	61.1 (58.8–63.4)	1.31 (1.13–1.52)	0.92 (0.88–0.96)
≥ 20%	16.3 (14–18.8)	89.8 (88.0–91.3)	52.3 (46.5–58.1)	60.8 (58.6–62.9)	1.59 (1.29–1.96)	0.93 (0.91–0.96)
≥ 25%	9.7 (7.9–11.8)	95.5 (94.3–96.5)	60.0 (51.8–67.7)	60.5 (58.4–62.5)	2.17 (1.59–2.96)	0.95 (0.93–0.97)
≥ 30%	4.9 (3.7–6.5)	98.3 (97.5–98.9)	67.1 (54.8–77.6)	59.9 (57.9–61.9)	2.95 (1.81–4.83)	0.97 (0.95–0.98)
Smaller twins						
≥ 15%	32.8 (30.0–35.7)	84.5 (82.3–86.4)	63.8 (59.6–67.8)	60.1 (57.8–62.4)	2.11 (1.81–2.46)	0.80 (0.76–0.83)
≥ 20%	20.7 (18.3–23.3)	94.0 (92.5–95.2)	74.2 (68.7–79)	58.7 (56.5–60.8)	3.44 (2.69–4.40)	0.84 (0.82–0.87)
≥ 25%	12.0 (10.1–14.1)	97.9 (96.9–98.6)	82.6 (75.5–88.0)	57.1 (55–59.2)	5.68 (3.78–8.54)	0.90 (0.88–0.92)
≥ 30%	6.3 (4.9–7.9)	99.8 (99.3–99.9)	95.7 (87.2–98.9)	56.1 (54–58.1)	26.77 (8.44–84.85)	0.94 (0.93–0.95)

## Discussion

Based on the retrospective data of 2348 twin pairs, we found that birthweight discordance, regardless of the previously suggested thresholds (15, 20, 25 and 30%), was associated with NICU admission. The risks of respiratory morbidity as well as those of composite outcome increased when a cutoff of 20% or greater was chosen. ROC analyses showed that the performance of BWD in predicting neonatal adverse outcomes was poor. Among these selected cutoffs, BWD ≥30% had the best accuracy for predicting NICU admission among twin pregnancies.

Prior studies evaluated perinatal morbidity and mortality between concordant and discordant twins, despite the selection of various cutoffs [6, 11, 15, 16, 19, 20, 23–28]. Finding an optimal threshold of discordant growth might benefit decision-making regarding the clinical management of twin gestations. Several limitations, including a lack of chorionicity data, small sample size and use of EFW, impede reaching a consensus on the optimal cutoff in clinical practice. In the current study, using the retrospective data of 2348 twin pairs, we tried to evaluate selected cutoffs from the perspective of early neonatal outcomes. We found increased risks of NICU admission in twins with BWD despite the selected cutoffs. This finding was similar to that of D′ Antonio et al. [19]. However, they found no significant risk of NICU admission among twins with BWD ≥15% in an adjusted model. One possible explanation could be the more stringent inclusion criteria for gestational age at delivery in D′ Antonio et al.’s study compared with ours (34 weeks vs. 28 weeks). In addition, this risk increased with the increasing cutoff, which was further confirmed by the RSC models. These results might be useful for risk stratification among twin pregnancies in clinical practice. In the current study population, twins with severe BWD had a higher incidence of SGA. The inherent risk of SGA fetuses could contribute to this situation. After we controlled for SGA, the risks remained significant but lower when compared with those unadjusted for SGA. This result was supportive of the previous finding of Amaru et al. [27]. These researchers found that even among appropriate for gestational age (AGA) twins, discordant birthweight (≥20%) was associated with NICU admission. Another cohort of 895 dichorionic and 250 monochorionic AGA twin pairs, however, showed that the risk of NICU admission was not increased in discordant (≥20%) dichorionic twins (RR, 1.5; 95% CI, 1.0–2.3) but was increased in discordant monochorionic twins (RR, 2.9; 95% CI, 2.0–4.3) [29]. Unfortunately, the researchers did not take the intertwin correlation into account. We found that the ORs of NICU admission were higher among MCDA twins than DCDA twins. This result may be explained by the different pathophysiology of discordant growth between MCDA and DCDA twins, since the former have a shared placenta influencing both twin fetuses.

We also found increased respiratory morbidities, including NRDS and ventilator support, among BWD (≥20% or greater) twins. This finding was inconsistent with some studies [6, 11, 15, 16, 26, 28] but supported by others [27, 30]. This difference is probably due to significantly earlier gestational age among the twins with severe BWD given that they were usually associated with gestational hypertensive disorder [15, 16]. Management of discordant twins is challenging. Increased surveillance and early delivery would benefit the survival of discordant twins since discordant growth was a contributor to intrauterine death in twins [9, 31, 32]. Preterm birth, however, remains the priority of adverse factors of neonatal prognosis. In this regard, the timing of delivery deserves to be further studied. Consistent with previous studies [6, 19, 20], we defined a composite outcome containing major morbidity and neonatal death and found increased risk among twins with BWD (≥20% or greater). Given that the current literature lacks a validated composite index to measure neonatal morbidity, these results should be interpreted with caution.

Although BWD was associated with a significant increase in adverse neonatal outcomes, the ROC curve analysis obtained from our data suggested that BWD was unable to serve as a single predictor in clinical use (AUROC: 0.569; 95% CI: 0.526–0.612). Similarly, Vergani et al. [20] reported an AUROC of 0.655 ± 0.033 among preterm twins. D’Antonio et al. [19] reported an AUROC of 0.58 (95% CI: 0.53–0.63) among pregnancies beyond 34 weeks of gestation. Jahanfar et al. [5] reported an AUROC of 0.54 (95% CI: 0.51–0.56) among pregnancies beyond 20 weeks of gestation. Among these selected thresholds, a threshold of ≥30% was moderately predictable of NICU admission in twin gestations, suggesting that this cutoff was clinically practical. Interestingly, the results stratified by chorionicity showed that the AUROC was greater in MCDA twins than in DCDA twins (0.676 vs. 0.545), which suggested BWD was more predictable for NICU admission among MCDA twins. Moreover, the difference between AUROCs suggested that BWD was more predictable for NICU admission among the smaller twins than it was among the larger twins.

One of the strengths of the current study was large-sized birthweight data with chorionicity information. Moreover, the use of GEE models accounting for intertwin correlation enables us to obtain robust results on associations between BWD and neonatal outcomes. Several limitations, nevertheless, should be considered when interpreting the current results. First, the retrospective nature of the study represents its main limitation. Birthweight was used to prevent the missing diagnosis of discordant twins. A recent prospective study using EFW by sonography suggested that the percentage of dichorionic twins exceeding a fixed discordance cutoff increased as gestation advanced [33]. Given this, caution should be taken when generalizing current results to those populations using estimated fetal weight based on sonography. Whether twins with EFW discordance at a lower level should be closely monitored deserves to be further discussed. Additionally, we were unable to exclude the possibility of confounders, such as fetuses with abnormal Doppler and the use of antennal corticosteroids, due to the lack of this information. The second limitation was the failure to detect differences in individual major morbidities due to the smaller number of events. Defining a composite measure might lack validity despite the increased power of analysis. Third, this study was based on a single-center database, therefore current results might be less generalized. Fourth, although gestational age was included for adjustment in the models, we could not provide stratified results according to gestational age since most of early preterm births attended NICU.

## Conclusions

Although BWD is independently associated with adverse neonatal outcomes among twin gestations, it could not serve as a single predictor for neonatal outcomes given the weak discriminative ability to predict neonatal outcomes. A cutoff of 30% is more practical for risk stratification among twin gestations.

## Supplementary Information

**Additional file 1.**

## Data Availability

The datasets used or analyzed in current study are available from the corresponding author on reasonable requests.

## Abbreviations

BWD: Birthweight discordance
NICU: Neonatal intensive care unit
NRDS: Neonatal respiratory distress syndrome
GEE: Generalized estimated equation
RCS: Restrictive cubic spline
ROC: Receiver operating characteristic
EFW: Estimated fetal weight
NICE: National Institute for Health and Care Excellence
TTTS: Twin-to-twin transfusion
ART: Assisted reproductive technology
SGA: Small for gestational age
HIE: Hypoxic-ischemic encephalopathy
BPD: Bronchopulmonary dysplasia
NEC: Necrotizing enterocolitis
ICH: Intracranial hemorrhage
ANOVA: Analysis of variance
OR: Odds ratios
CI: Confidence interval
LR +: Positive likelihood ratio
LR-: Negative likelihood ratio
AUROC: Area under the ROC curve
