# Supplementary material for: Assessment of different thresholds of birthweight discordance for early neonatal outcomes: retrospective analysis of 2348 twin pregnancies
Source: BMC Pregnancy Childbirth. 2022 Feb 1;22:93. doi: 10.1186/s12884-022-04417-4 (PMC8808974; doi:10.1186/s12884-022-04417-4)
Supplement: Supplementary file 1 — Additional file 1. [file 12884_2022_4417_MOESM1_ESM.docx]

| Table S1. Association between neonatal outcomes and birthweight discordance by different cutoffs and chorionicity. | | | | | | | | | |
| --- | --- | --- | --- | --- | --- | --- | --- | --- | --- |
| Outcomes | MCDA twins (n=804) | | | |  | DCDA twins (n=3892) | | | |
|  | Crude OR | *P*-value | Adjusted OR^a^ | *P*-value |  | Crude OR | *P*-value | Adjusted OR^a^ | *P*-value |
| NICU admission |  |  |  |  |  |  |  |  |  |
| ≥15% | 3.75 (2.34-6.03) | <0.001 | 2.75 (1.57-4.81) | <0.001 |  | 1.71 (1.41-2.07) | <0.001 | 1.38 (1.07-1.77) | 0.012 |
| ≥20% | 5.13 (2.66-9.92) | <0.001 | 3.11 (1.47-6.61) | 0.003 |  | 2.25 (1.76-2.89) | <0.001 | 1.65 (1.21-2.26) | 0.002 |
| ≥25% | 9.74 (3.22-29.49) | <0.001 | 5.63 (1.80-17.59) | 0.003 |  | 3.03 (2.14-4.29) | <0.001 | 1.87 (1.22-2.87) | 0.004 |
| ≥30% | 13.07 (2.80-60.96) | <0.001 | 4.48 (1.03-19.45) | 0.046 |  | 4.80 (2.63-8.77) | <0.001 | 2.02 (1.04-3.94) | 0.038 |
| NRDS |  |  |  |  |  |  |  |  |  |
| ≥15% | 2.01 (1.04-3.91) | 0.039 | 1.64 (0.70-3.87) | 0.254 |  | 1.14 (0.80-1.62) | 0.461 | 0.92 (0.57-1.46) | 0.710 |
| ≥20% | 2.19 (1.03-4.68) | 0.043 | 1.72 (0.65-4.55) | 0.274 |  | 1.66 (1.11-2.48) | 0.014 | 2.06 (1.21-3.50) | 0.008 |
| ≥25% | 3.09 (1.29-7.42) | 0.012 | 2.77 (0.93-8.24) | 0.067 |  | 2.12 (1.29-3.47) | 0.003 | 1.81 (0.93-3.52) | 0.080 |
| ≥30% | 3.19 (1.15-8.83) | 0.026 | 2.72 (0.80-9.23) | 0.109 |  | 2.48 (1.21-5.06) | 0.013 | 2.02 (0.81-5.17) | 0.132 |
| Ventilator support |  |  |  |  |  |  |  |  |  |
| ≥15% | 1.57 (0.85-2.90) | 0.151 | 1.00 (0.46-2.16) | 0.994 |  | 1.36 (0.99-1.87) | 0.060 | 1.17 (0.77-1.76) | 0.466 |
| ≥20% | 1.40 (0.66-2.96) | 0.384 | 0.65 (0.25-1.68) | 0.371 |  | 1.64 (1.12-2.40) | 0.011 | 1.86 (1.15-3.02) | 0.012 |
| ≥25% | 1.74 (0.70-4.29) | 0.232 | 0.98 (0.33-2.90) | 0.971 |  | 2.25 (1.42-3.55) | 0.001 | 1.89 (1.04-3.44) | 0.037 |
| ≥30% | 1.46 (0.47-4.50) | 0.509 | 0.75 (0.21-2.68) | 0.657 |  | 3.40 (1.83-6.34) | <0.001 | 3.72 (1.71-8.08) | 0.001 |
| Composite outcome |  |  |  |  |  |  |  |  |  |
| ≥15% | 1.74 (0.54-5.59) | 0.354 | 1.32 (0.37-4.73) | 0.672 |  | 1.39 (0.82-2.37) | 0.226 | 1.27 (0.72-2.25) | 0.414 |
| ≥20% | 1.33 (0.31-5.80) | 0.702 | 0.72 (0.14-3.85) | 0.705 |  | 1.86 (1.02-3.41) | 0.044 | 2.19 (1.14-4.22) | 0.018 |
| ≥25% | 1.62 (0.28-9.25) | 0.590 | 1.19 (0.16-9.09) | 0.867 |  | 3.08 (1.59-5.96) | 0.001 | 2.85 (1.36-6.01) | 0.006 |
| ≥30% | 2.49 (0.43-14.52) | 0.311 | 2.42 (0.33-18.04) | 0.387 |  | 4.57 (1.97-10.63) | <0.001 | 5.52 (2.10-14.5) | 0.001 |
| a, adjusted for nulliparity, use of ART, gestational age and SGA status. | | | | | | | | | |

| Table S2. Association between neonatal outcomes and birthweight discordance by different cutoffs and larger or smaller twins. | | | | | | | | | |
| --- | --- | --- | --- | --- | --- | --- | --- | --- | --- |
| Outcomes | Larger twins (n=2348) | | | |  | Smaller twins (n=2348) | | | |
|  | Crude OR | *P*-value | Adjusted OR^a^ | *P*-value |  | Crude OR | *P*-value | Adjusted OR^a^ | *P*-value |
| NICU admission |  |  |  |  |  |  |  |  |  |
| ≥15% | 1.43 (1.18-1.73) | <0.001 | 1.16 (0.91-1.48) | 0.220 |  | 2.65 (2.17-3.23) | <0.001 | 1.72 (1.32-2.25) | <0.001 |
| ≥20% | 1.70 (1.33-2.17) | <0.001 | 1.51 (1.11-2.05) | 0.009 |  | 4.08 (3.10-5.36) | <0.001 | 2.55 (1.77-3.68) | <0.001 |
| ≥25% | 2.29 (1.65-3.20) | <0.001 | 1.91 (1.26-2.89) | 0.002 |  | 6.32 (4.14-9.65) | <0.001 | 2.43 (1.4-4.22) | 0.002 |
| ≥30% | 3.06 (1.84-5.07) | <0.001 | 2.46 (1.31-4.59) | 0.005 |  | 28.49 (8.94-90.85) | <0.001 | 10.8 (2.53-46.04) | 0.001 |
| NRDS |  |  |  |  |  |  |  |  |  |
| ≥15% | 1.32 (0.96-1.81) | 0.091 | 1.15 (0.75-1.77) | 0.514 |  | 1.25 (0.90-1.72) | 0.178 | 0.73 (0.45-1.19) | 0.206 |
| ≥20% | 1.82 (1.26-2.63) | 0.001 | 2.21 (1.36-3.59) | 0.001 |  | 1.70 (1.17-2.46) | 0.005 | 1.39 (0.77-2.48) | 0.272 |
| ≥25% | 2.25 (1.44-3.53) | <0.001 | 2.12 (1.17-3.84) | 0.013 |  | 2.37 (1.52-3.69) | <0.001 | 1.52 (0.75-3.06) | 0.244 |
| ≥30% | 2.72 (1.48-4.97) | 0.001 | 2.60 (1.21-5.60) | 0.015 |  | 2.72 (1.48-4.97) | 0.001 | 1.59 (0.67-3.80) | 0.296 |
| Ventilator support |  |  |  |  |  |  |  |  |  |
| ≥15% | 1.44 (1.07-1.96) | 0.018 | 1.28 (0.84-1.95) | 0.256 |  | 1.35 (0.99-1.85) | 0.058 | 0.86 (0.53-1.41) | 0.555 |
| ≥20% | 1.56 (1.08-2.25) | 0.017 | 1.60 (0.97-2.64) | 0.067 |  | 1.62 (1.12-2.35) | 0.010 | 1.27 (0.70-2.32) | 0.430 |
| ≥25% | 1.92 (1.22-3.01) | 0.005 | 1.51 (0.81-2.82) | 0.198 |  | 2.39 (1.55-3.70) | <0.001 | 1.65 (0.80-3.36) | 0.173 |
| ≥30% | 2.22 (1.20-4.13) | 0.011 | 1.84 (0.81-4.16) | 0.143 |  | 3.41 (1.94-6.00) | <0.001 | 2.69 (1.15-6.33) | 0.023 |
| Composite outcome |  |  |  |  |  |  |  |  |  |
| ≥15% | 1.35 (0.77-2.36) | 0.288 | 1.26 (0.63-2.50) | 0.514 |  | 1.53 (0.87-2.70) | 0.142 | 1.21 (0.58-2.50) | 0.614 |
| ≥20% | 1.50 (0.77-2.92) | 0.229 | 1.62 (0.72-3.65) | 0.240 |  | 2.08 (1.11-3.91) | 0.023 | 2.10 (0.89-4.95) | 0.089 |
| ≥25% | 2.16 (1.01-4.61) | 0.048 | 1.85 (0.72-4.76) | 0.199 |  | 3.57 (1.81-7.03) | <0.001 | 3.37 (1.24-9.13) | 0.017 |
| ≥30% | 2.32 (0.82-6.58) | 0.114 | 2.34 (0.65-8.40) | 0.191 |  | 5.87 (2.67-12.92) | <0.001 | 8.12 (2.39-27.62) | 0.001 |
| a, adjusted for nulliparity, use of ART, gestational age, chorionicity and SGA status. | | | | | | | | | |


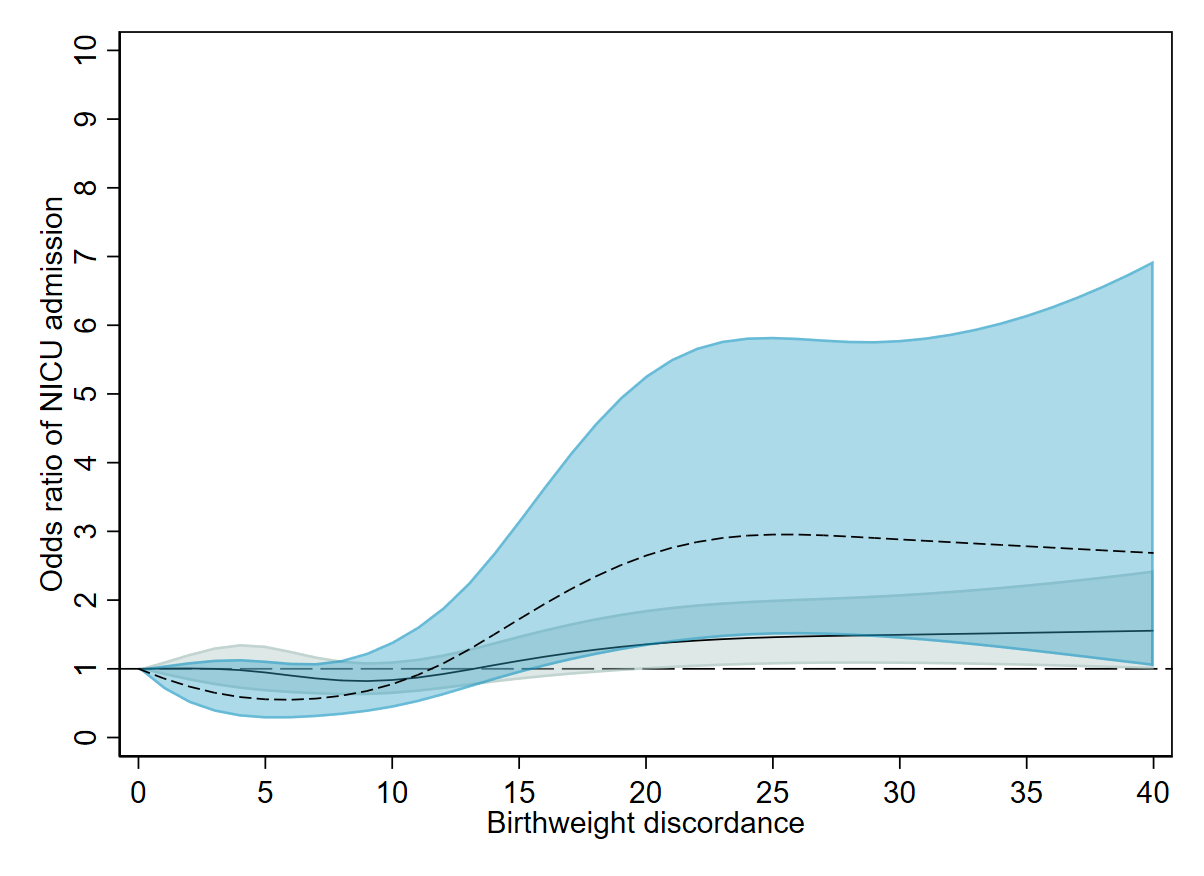


**Figure S1**. The dose-response relationships between birthweight discordance and NICU admission in MCDA and DCDA twins. Long dashed lines represent the reference line (OR=1.0); the solid line represents the ORs and the gray band represents the 95% confidential intervals in DCDA twins; the short dash represents the ORs and the blue band represents the 95% confidential intervals in MCDA twins.


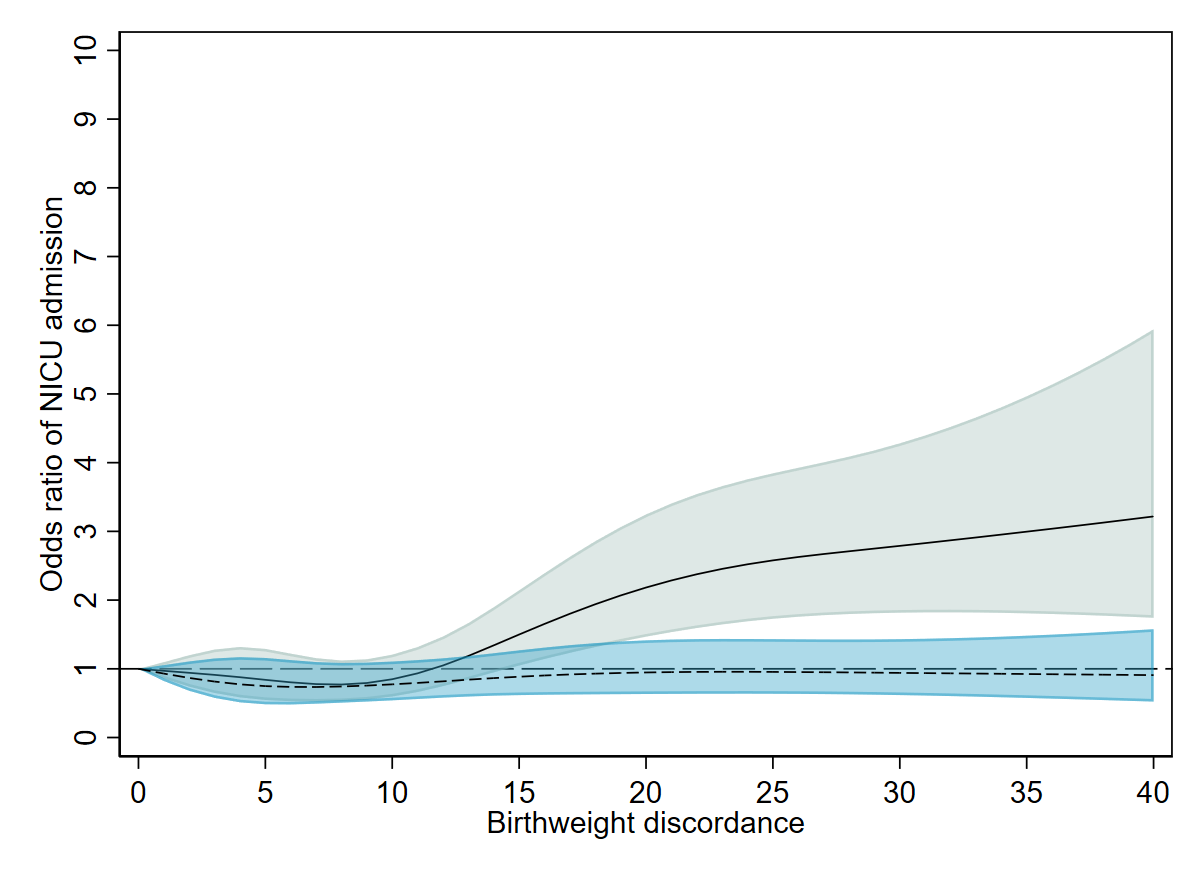


**Figure S2**. The dose-response relationships between birthweight discordance and NICU admission in smaller and larger twins. Long dashed lines represent the reference line (OR=1.0); the solid line represents the ORs and the gray band represents the 95% confidential intervals in smaller twins; the short dash represents the ORs and the blue band represents the 95% confidential intervals in larger twins.


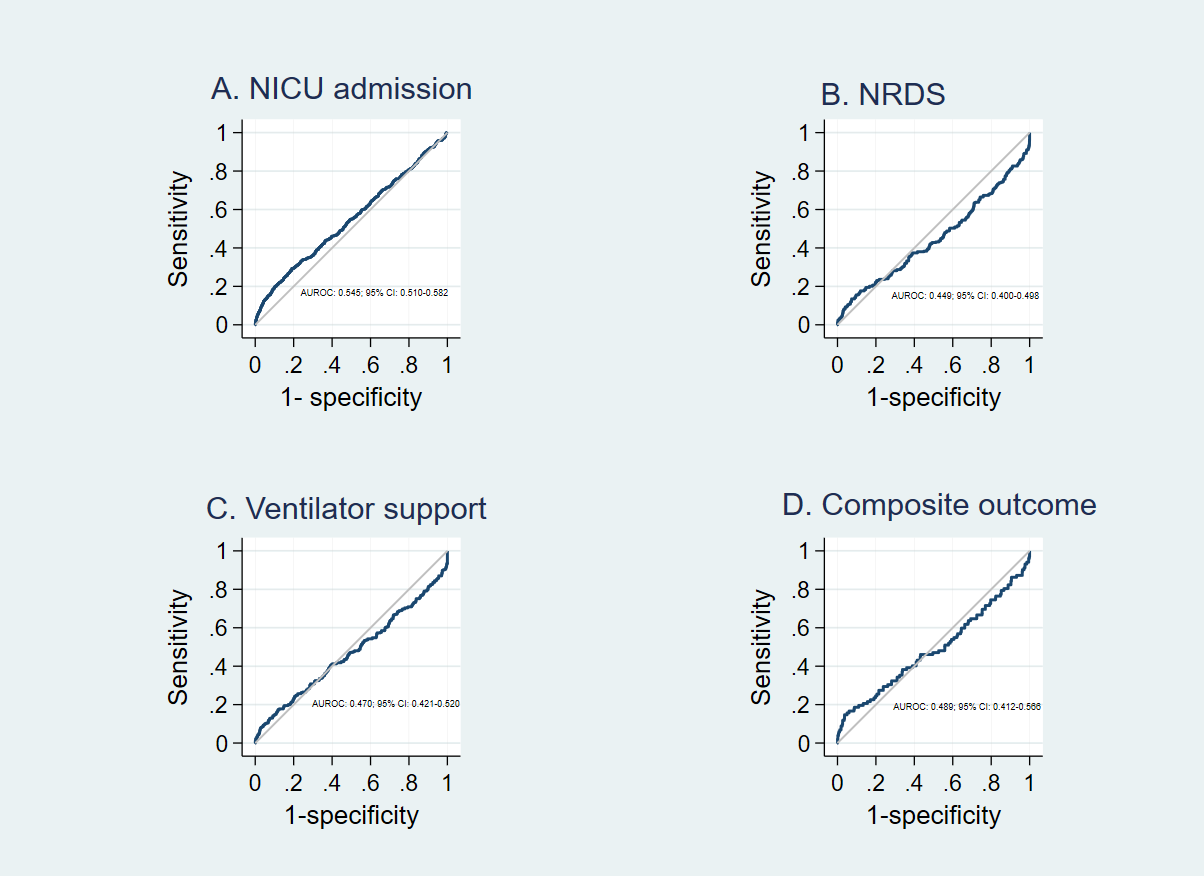


**Figure S3**. ROC curve analysis of birthweight discordance for the prediction of neonatal outcomes in DCDA twins.


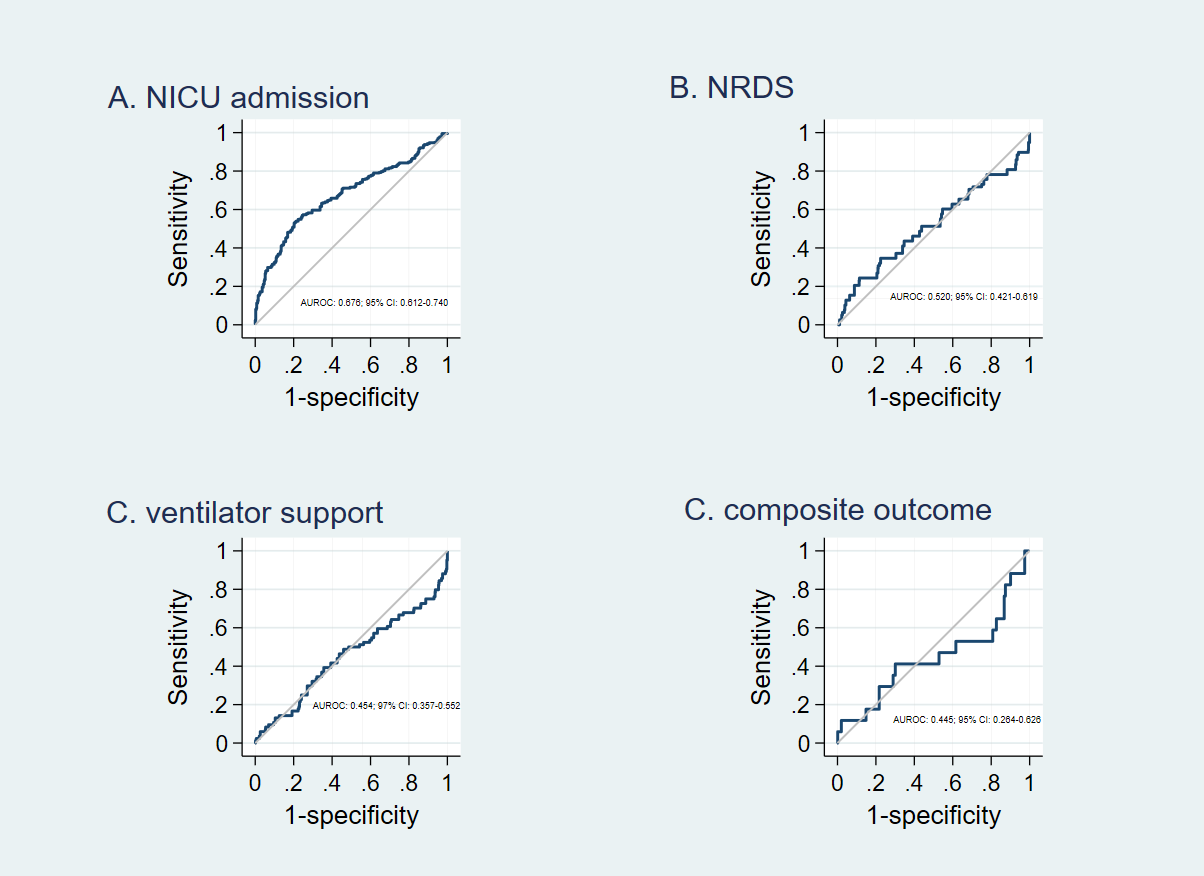


**Figure S4**. ROC curve analysis of birthweight discordance for the prediction of neonatal outcomes in MCDA twins.


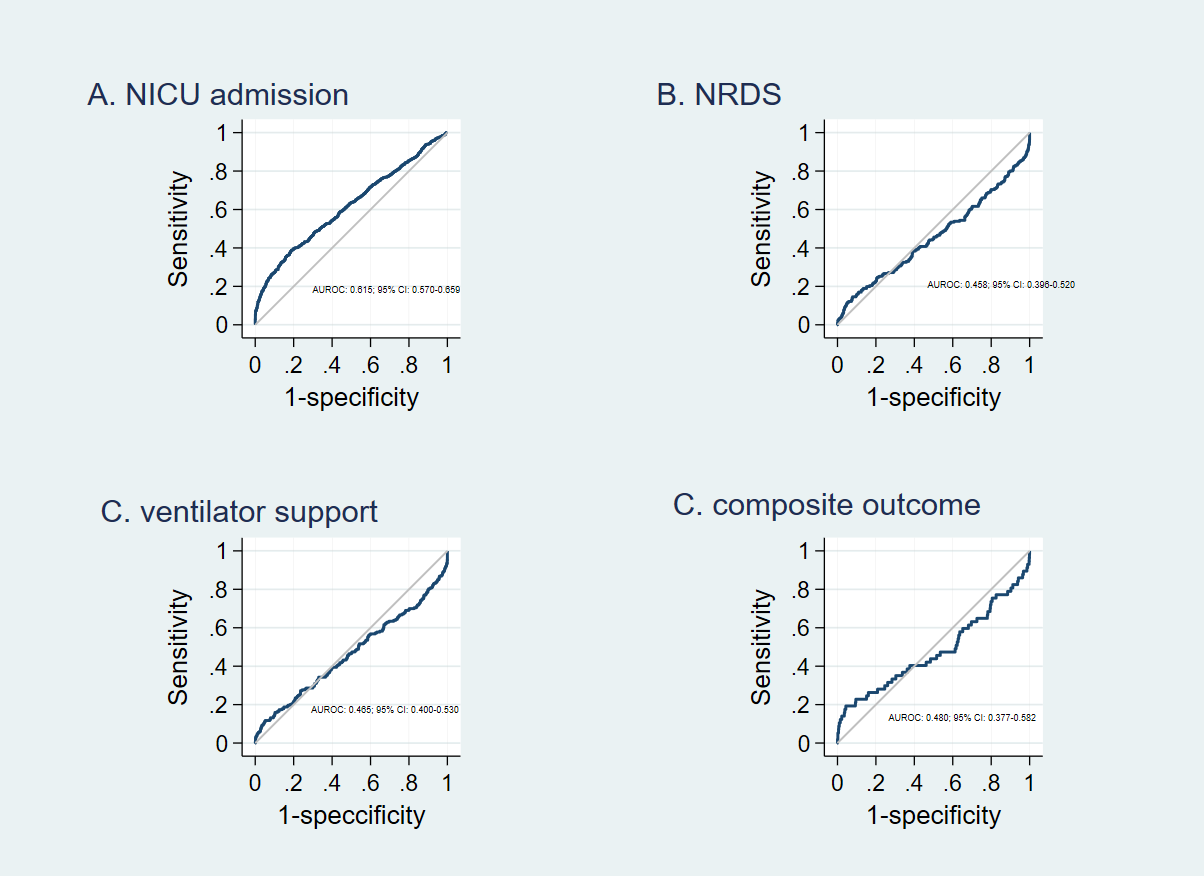


**Figure S5**. ROC curve analysis of birthweight discordance for the prediction of neonatal outcomes in smaller twins.


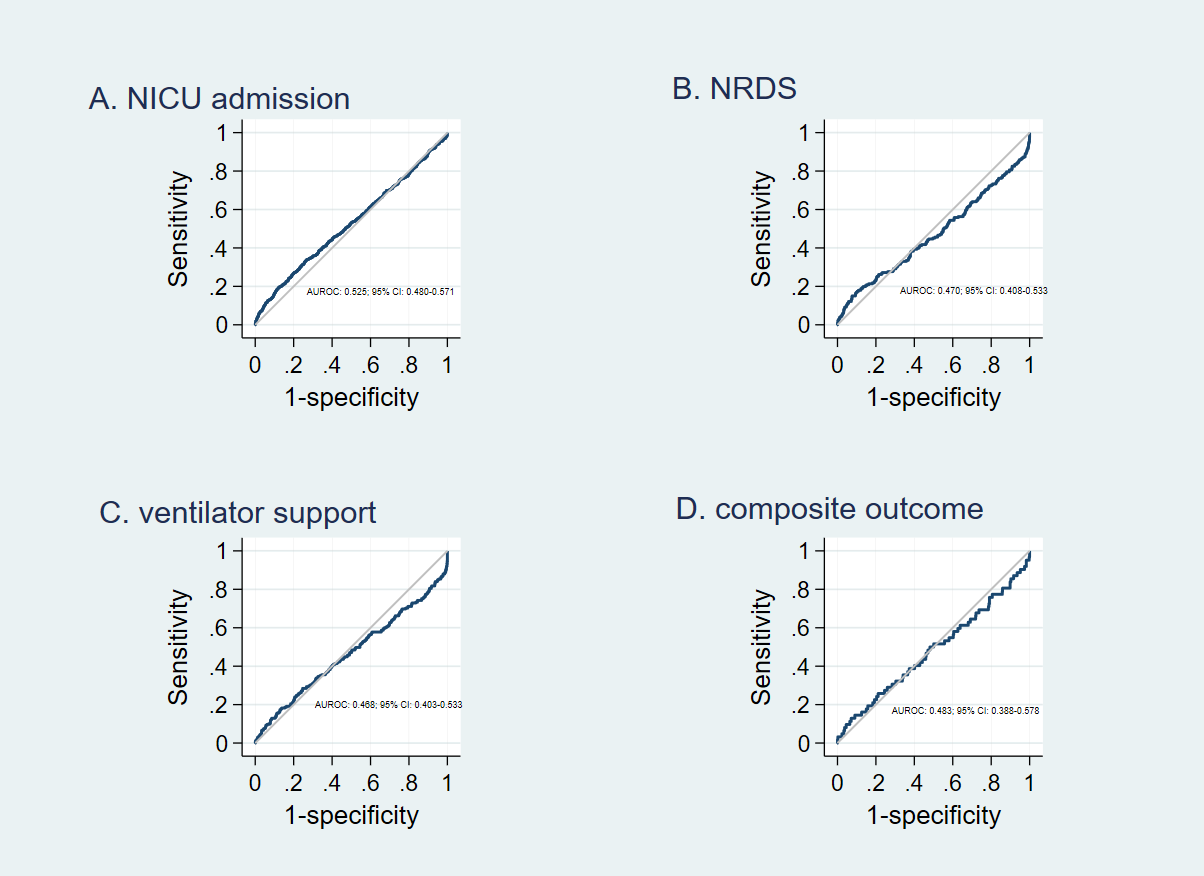


**Figure S6**. ROC curve analysis of birthweight discordance for the prediction of neonatal outcomes in larger twins.
